# Kraft pulp mill dregs and grits as permeable reactive barrier for removal of copper and sulfate in acid mine drainage

**DOI:** 10.1038/s41598-020-60780-2

**Published:** 2020-03-05

**Authors:** Rogério M. P. Farage, Margarida J. Quina, Licínio Gando-Ferreira, Cláudio M. Silva, José João L. L. de Souza, Caio M. M. E. Torres

**Affiliations:** 1 Departamento de Engenharia Florestal, Universidade Federal de Viçosa, Viçosa, Brazil; 2 CIEPQPF- Chemical Process Engineering and Forest Products Research Centre, Department of Chemical Engineering, University of Coimbra, Coimbra, Portugal; 3 Departamento de Solos, Universidade Federal de Viçosa, Viçosa, Brazil

**Keywords:** Environmental sciences, Environmental chemistry, Environmental impact

## Abstract

Mining is an essential human activity, but results in several environmental impacts, notably the contamination of ground and surface water through the presence of toxic substances such as metals and sulfates in mine drainage. Permeable reactive barriers (PRB) have been applied to remediate this environmental impact, but the high costs associated with the maintenance of this system are still a challenge. The main objective of this study was to evaluate the use of kraft pulp mill alkaline residues, known as dregs and grits, as material for PRB, and to determine their capacity for retaining copper and sulfate. The work was carried out in laboratory adsorption kinetics assays, batch assays and column tests. Tests for elemental characterization, point of zero charge, acid neutralization capacity, total porosity, bulk density and moisture of the dregs and grits were conducted. The results showed high retention of Cu due to a chemical precipitation mechanism, notably for dregs (99%) at 5 min in adsorption kinetics. The grits presented similar results after 180 min for the same assay. Sulfate retention was effective at pH below 5, with an efficiency of 79% and 89% for dregs and grits, respectively. Dregs presented the best results for acid drainage remediation, notably with a solid:liquid (S:L) ratio of 1:10.

## Introduction

Acid mine drainage (AMD) is caused by the oxidation of sulfur-bearing materials in the presence of oxygen and water, resulting in sulfate and metals solubilization and forming acidic solutions^1–4^. Active and inactive mining activities for metals, such as copper, lead, zinc, gold, silver and uranium, provoke potential degradation of the environment by the presence of different contaminants, particularly due to metals and sulfates in acid drainage^5^. Indeed, the acid drainage formation is an important environmental aspect of mining practices. The sulfate concentrations may reach 2,400 to 20,800 mg/L^6,7^, well above the limit of 250 mg/L established by the World Health Organization^8^. It might cause damage not only to human health but also to the environment, and depreciation of structures and equipment^2,9^, resulting in high costs for control and management by the mining companies^2,10^. Copper concentration may be in the range from 77 to 615 mg/L, with higher values in the Cu mines^1,11^. Other metals such as Al, Mn, Fe, Mg, and Zn are also commonly found in AMD in these mines^10,12^.

AMD remediation can be actively accomplished off-site with the pumping of the contamination plume to a treatment plant, or passively on-site with the use of permeable reactive barriers (PRB), with lower installation and maintenance costs. It can be carried out with the flow directed to the system, by the Funnel model, or the grid-type, by the installation of a perpendicular barrier to the contamination plume^13,14^. The PRB was first proposed in Canada in the 1990s^13,15^, with different materials used as adsorbent media, from single barriers with only one material to composites of different materials, or multiple sequential barriers^16,17^. Materials such as activated carbon, zeolites, zero-valent iron and powder from cement furnaces mixed with quartz sand have been studied for application in PRBs^18–21^. The selection of adsorbent materials is determined primarily by the types of contaminants to be removed, the desired levels of removal, the costs, the shelf life of the materials and their regeneration potential^22^. The use of industrial wastes or by-products, such as dregs generated in pulp and paper mills, has been studied due to the low cost and the opportunity to increase the lifespan of these materials, reducing the environmental pressure on final disposal methods and natural resource exploitation^23,24^. Those measures are in accordance with the principles of the Circular Economy, a policy with increasing application in the countries of the European Union.

In addition, PRBs can be applied for the neutralization of pH and remediation of organic contaminants, especially by mechanisms of adsorption, precipitation, and biological degradation. They have as their main advantage the potential for on-site treatment, and as a disadvantage the lifespan of the adsorbent materials and the maintenance costs^13^. High-efficiency methods for the removal of sulfates and metals in acid mine drainage (AMD) have been proposed using individual or sequential PRBs filled with limestone and BaCO_3_, respectively, resulting in complete removal of metals and more than 70% of the sulfate, by precipitation and adsorption mechanisms^10^.

Dregs and grits originating in the recovery of the chemical liquor from kraft pulp mills have usually been sent to industrial landfills. Both are alkaline by-products with pH in the range of 10.5 to 13^25^. Dregs are mainly composed of sodium and calcium carbonates (Na_2_CO_3_ and CaCO_3_) and sodium sulfide (Na_2_S), and are generated from the separation of CaCO_3_ and CaO in the green liquor. Grits are formed mainly by CaCO_3_ and CaO which did not react in the slaker^26–29^. Those characteristics potentiate the mechanisms of chemical precipitation and adsorption, suggesting an attractive application in the retention of metallic and sulfate ions^9,30–32^. Efficient removal of metals such as Cd, Cr, Cu, Ni, Zn and Pb in solution, notably by precipitation, are mainly attributed to calcium carbonate^33^, with 93% average removal of Fe and Mn in aquifers contaminated by landfill leachate in the USA^34^. Dregs and grits were studied to serve as intermediate covering in urban solid wastes landfills in order to retain metals by precipitation avoiding contamination of the leachate and to prevent the presence macro and micro vectors in the landfill site^35^. It is important to note that metals such as Cd, Cr, Cu, Ni, and Pb have been identified in samples of dregs and grits, but in lower concentrations than in soil background^26,36,37^. In fact, their applications in soil did not cause leachates with characteristics above the limits established by the Council of the European Economic Community in their Directive 86/278/EEC, and the values found were similar to the application of commercial limestone^25^.

This study proposes the use of kraft pulp mill dregs and grits as reactive materials in PRBs. It is expected the removal of copper and sulfate ions in AMD through precipitation and/or adsorption mechanisms.

## Material and Methods

### Material and samples preparation

Samples of dregs and grits (about 5 kg of each) were collected in a pulp mill in the Center region of Portugal. Both samples were dried at 40 °C until constant weight was achieved^38^, then ground and sifted to produce fragments of 0.5 to 1.0 mm^10,39^. A solution of copper and sulfate was prepared with anhydrous Na_2_SO_4_ and CuSO_4_.

A synthetic solution was used in the experiment to assure that all variables and the concentration of each heavy metal was controlled.

### Characterization of dregs and grits

The adsorbent materials (dregs and grits) were characterized by Energy Dispersive X-ray Fluorescence (EDXRF), using a NEX CG Rigaku Spectrometer.

The bulk porosity of both materials was determined by weighing a graduated test tube filled with each material under analysis and then saturated with kerosene^10^. Initially, the tube was filled with 10 mL of powdered material and weighted. Subsequently, kerosene was added until the material reached saturation, and the tube was weighted again. The total porosity, ε_b_, was determined by Eq.(1)

1 $${\varepsilon }_{b}=\frac{{M}_{kerosene}/{{\rm{\rho }}}_{kerosene}}{V}100$$

Where M_kerosene_ is the mass of kerosene in the tube filled with particulate material (g), ρ_kerosene_ is the density of the kerosene (g/mL) and V is the volume of the graduated test tube (mL).

Moisture was measured as the loss in weight, after samples were dried at 106 ° C until reaching a constant weight.

The volatile solids were determined according to Standard Methods for the Examination of Water and Wastewater^40^.

The bulk density was measured by pouring a sample of known mass into a graduated test tube. The recipient was filled and weighed 5 times, and the average value given in g/cm³.

The pH and electrical conductivity (EC) were determined using a pH meter and a conductivity meter.

The buffering capacity (or acid neutralization capacity – ANC) of the dregs and grits was determined using the batch titration method, with 0.75 g of dregs and grits to 20 mL of deionized water, being stirred for 24 h. The titrations were conducted by adding 0 to 16 mL of HCl 1 M^41^. Following this procedure, the buffer capacity was measured over a pH variation in different solid:liquid (S:L) ratios (1:10, 1:20, 1:50, 1:100 and 1:1000), stirring for 24 h at 100 rpm.

The pH corresponding to the point of zero charge, pH_pzc_, for the dregs and grits was determined by adding 0.1 g of each solid material to 20 mL of KNO_3_ solution (0.01 M) in a plastic vial of 50 mL and shaking for 24 h. Plastic vials were prepared, with initial pH adjusted from 2 to 12. This range of pH was covered by a different acidity in each vial. The pH_pcz_ was determined when pH∆ (pH_f_ - pH_i_) was zero. The pH_pcz_ corresponds to the pH of a solution for which the particles surface charge is neutral. Thus, if the pH is smaller than pH_pcz_, the solid particles are positively charged (attracting anions), while in the opposite situation, the materials are negatively charged, attracting cations^42–44^.

### Column tests

The column tests and assays were carried out at the laboratories of the Chemical Engineering Department of the University of Coimbra, Portugal.

**Figure 1:**
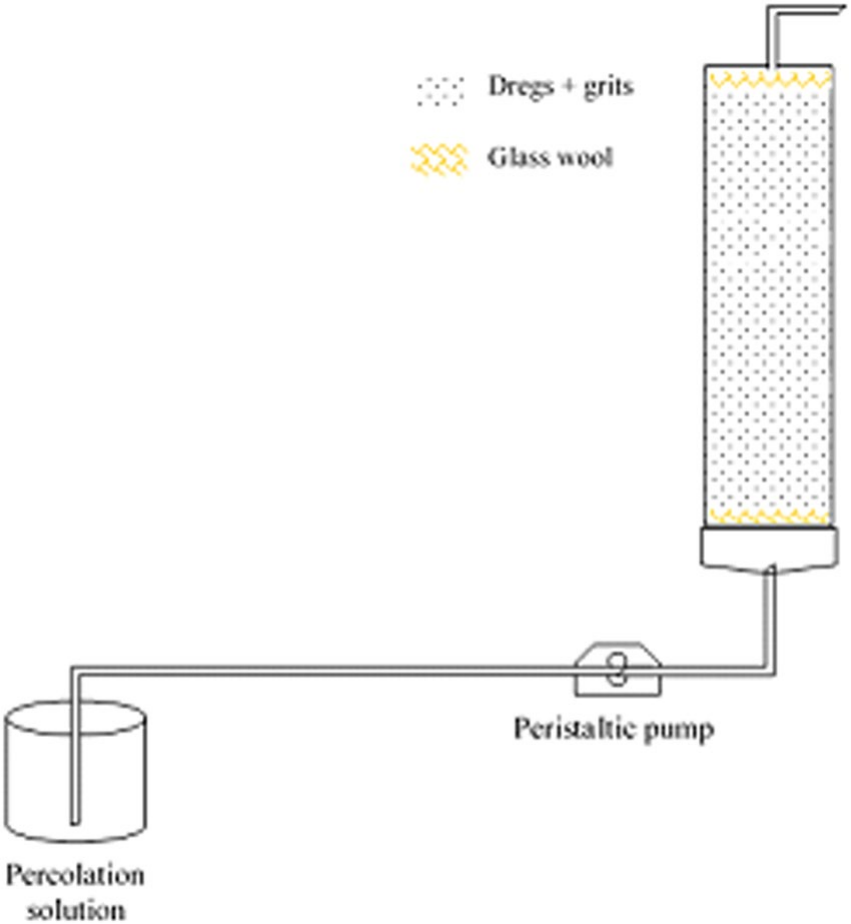
Adsorption column tests (scheme).

A column with a diameter of 32 mm and height of 100 mm was connected to a peristaltic pump, which introduced the solution into the column in an upward flow. A small layer of glass wool was placed at the bottom and in the top of the column to avoid losing the particulate materials (Fig. 1).

The percolate solution was prepared with anhydrous Na_2_SO_4_ and CuSO_4_ using distilled water^17^, with concentrations of 600 and 4,300 mg/L, respectively. These concentrations were set based on values usually found for AMD in copper mining^7,11^.

The column experiments were carried out in continuous-flow mode for 30 min with a total volume of a Cu and SO_4_ solution total volume of 200 mL, making a flow of 6.5 mL/min^3^ ^45^. The contact time was determined from the adsorption kinetics test, with 20 g of adsorbent material and 200 mL of Cu and SO_4_ solution, with a solid:liquid ratio of 1:10. The initial pH of the solution was 2.5 for both experiments with dregs and grits.

The concentrations of Cu present in the solution percolated through the adsorbent medium were determined by Energy-Dispersive X-Ray Fluorescence (EDXRF), using a NEX CG Rigaku Spectrometer. The determination of the sulfate concentration was accomplished using the titration method with Torina indicator solution, precipitating Na_2_SO_4_ with barium chloride to form barium sulfate^46^.

### Adsorption kinetics

The adsorption kinetics were assessed over a period of 180 min by taking samples at 5, 10, 20, 30, 60, 90 and 180 min. The suspension of 2 g in 20 mL of solution was maintained by constant stirring (100 rpm). The solution was prepared with 600 mg/L of Cu and 4,300 mg/L of SO_4_^2−^. The pH of the solution was adjusted to 2.5, according to the conditions common in AMD^10,47^. Two groups of tests were carried out. First, the pH was maintained below 2.5 by adding nitric acid. Nitric acid was used in order to avoid eventual precipitation of salts. Then, in the second set of tests, the pH was not controlled. These experiments were conducted using an S:L ratio equal to 1:10. Moreover, the removal efficiencies for Cu and SO_4_^2−^ were determined by using different S:L ratios.

## Results and Discussion

### Characterization of adsorption materials

Table 1 shows the concentration of calcium oxide in the kraft pulp mill dregs used in this study, which is within the typical values, 30–44%, in kraft pulp industries. Some variations are due to the efficiency of the liquor recovery process or to the origin of the processed wood^41^. Ca, Mg, Na, Fe, S, Mn, Si, Al, K, and P are the main inorganic elements present in the dry dregs^48^.

**Table 1:** Elemental contents (mg/Kg) measured in dregs and grits.

Oxide/Element	Dregs		Grits	
	Measured	[1;2]	Measured	[2]
CaO	425,000	300,000–440,000^a^	542,000	690,000
SiO_2_	8,300	10,630–26,570^a^	2,980	—
SO_3_	7,500	19,700–46,750^a^	1,390	1.4
MgO	50,000	34,830–84,750	5,800	2,160–9,450
Fe_2_O_3_	1,800	7,430–15,430	505	3,430–10,570
K_2_O	700	2,170–7,470	200	1,205–3,370
Al_2_O_3_	4,000	1,130–15,490	1,900	1,890–9,450
P_2_O_5_	7,100	4,120–9,620	2,800	3,310–10,990
Cl	153	**ND	42.0	—
Na	*14,700	29,800–144,400	*13,200	6,900–15,600
Ca	304,500	118,000–347,000	388,500	356,000–520,000

^1^Makitalo *et al*., 2014; ^2^Cabral *et al*., 2008; *Determined by Energy-Dispersive X-ray spectroscopy (EDX) on SHIMAZU’s EDX 1300 micro model spectrometer; **ND – not detected.

The potentially toxic metal concentrations are below or within the typical values (Table 2), and they show a similar characteristic, low solubility in water due to the high pH of dregs and grits^49^. The source of metal(loid)s in the dregs and grits is associated with their absorption from the soil by tree roots and their subsequent fixation in the wood, with the chemicals used in the process of cooking the wood chips, with the water used in the industrial processes, and as a product of the corrosion of the equipment^50^.

**Table 2:** Potentially toxic metals in dregs and grits.

ELEMENT	Dregs (mg/kg)					Grits (mg/kg)	
	Measured	[1]	[2]	[3]	[4]	Measured	[5]
Cd	ND	5.2	4.5	9.4	11	ND	4.75
Cr	15.3	56	39	118	160	0.54	12.4
Cu	54.9	81	129	102	330	16.3	4.6
Ni	32.5	189	175	84	80	ND	25
Pb	17.4	47	45	13	52	6.29	34
Zn	88.0	160	242	1,000	6,490	7.19	15

^1,5^Cabral *et al*., 2008; ^2^Modolo *et al*., 2010; ^3^Kinnarinen *et al*., 2016; ^4^Makela *et al*., 2016; ND: Not detected.

The total porosity of dregs and grits were 66% and 42%, respectively (Table 3). Porosities higher than 70% were found for dregs generated in a Swedish pulp mill^41^. The high porosity of this material has a direct positive relationship with the removal of contaminants in an aqueous media, mainly due to the intra-particle diffusion phenomenon^51,52^.

**Table 3:** Physical and chemical characterization of dregs and grits.

PARAMETERS	Dregs				Grits		
	Measured	[1]	[3]	[4]	Measured	[2]	[3]
Moisture (%)	31	48–65	—	—	6.5	—	16
pH	10.53	10–11	—	—	10.26	12–13	13
EC (mS/cm)	4.3	—	—	2.5	0.193	—	—
*VS (%)	3.5	—	8.3	—	0.17	—	2.4
**ρ_b_ (g/cm³)	0.61	0.67	—	0.44	1.26	—	—
ε (%)	66	73–82	—	—	42	—	—

^1^Makitalo *et al*., 2014; ^2^Cabral *et al*., 2008; ^3^Modolo *et al*., 2010; ^4^Pasandín *et al*., 2016.

*Volatile solids.

**Total porosity.

The size and homogeneity of the adsorbent particles are important with regards to the optimum performance of the PRB^21^. The plastic characteristic and low permeability of the dregs necessitate its mixing with another material^41^ in order to prevent the contamination plume from encountering a preferential flow of higher permeability outside the reactive barrier, a situation observed during the use of zero-valent iron as the only adsorbent medium^17,53^. The sintering mechanism can be used to adjust the particle size of adsorbent materials with their agglutination through the action of the temperature below the melting point^54^.

#### Acid neutralization capacity

**Figure 2:**
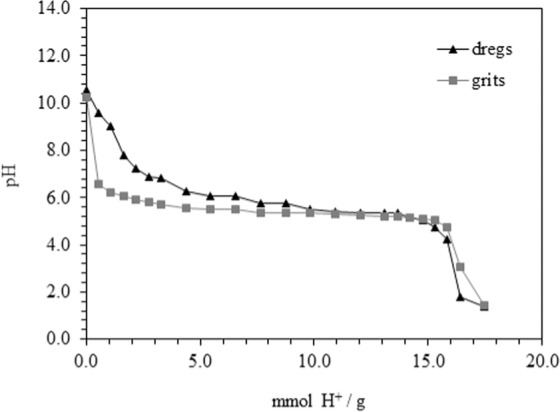
Acid neutralization capacity of dregs and grits.

Dregs and grits showed similar behavior regarding ANC, notably for pH lower than 7 (Fig. 2), with a major consumption of H^+^ between pH 7.0 and 5.0, suggesting the influence of the carbonates´ alkalinity^55^. For a similar study with dregs collected in Sweden, Makitalo^41^ also designated the same compounds as being alkaline. According to these assays, and using pH 4.5 as a reference, dregs and grits have an ANC of about 16 mmol H^+^/g. The high buffer capacity of the dregs is well known^56^, and it has been studied as an alternative liming material in soil^25^. The maintenance of the dregs´ alkaline condition, used as remediation of acid mine drainage in a long-term project, suggests a strong potential for this application^57^. Alkaline compounds such as carbonates and hydroxides in dregs and grits^25,49,50,55^ react with hydrogen ions, neutralizing or increasing the pH of the solution and removing metals through precipitation mechanisms^10,58^.

#### pH at the point of zero charge

**Figure 3:**
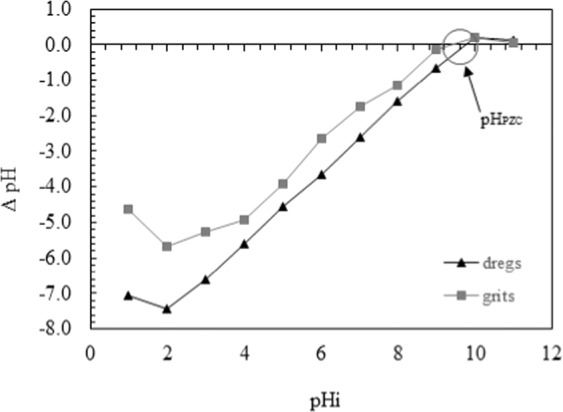
Determination of pH_pzc_ for dregs and grits.

**Figure 4:**
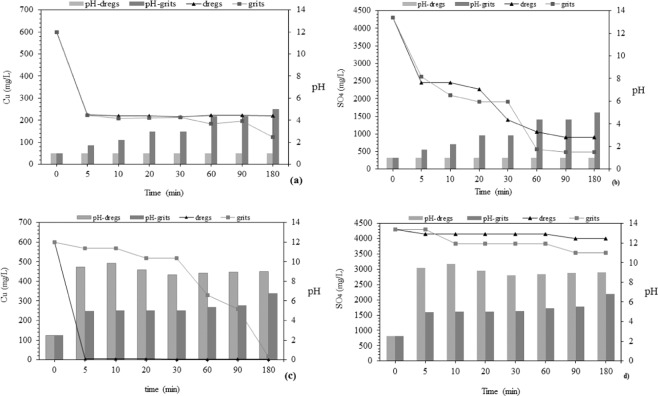
Adsorption kinetics of Cu and SO_4_ with pH control (**a**,**b**) and without pH control (**c**,**d**) for dregs and grits.

According to the results depicted in Fig. 3, the zero charge is achieved for dregs at pH_pzc_ 9.75 and for grits at pH_pzc_ 9.5. This condition suggests a potential for sulfate adsorption, such as presented in Fig. 4b, where more than 80% sulfate removal was achieved by other mechanisms, such as precipitation and ionic exchange^9^. High points of zero charge (8.5–11.0) were reported^59^, and the presence of CaCO_3_ in dregs and grits was pointed out as the phase responsible for this result. The knowledge of the surface charge is important for selecting the adsorption materials, according to the specific contaminants whose removal is desired^43^. The use of sequential permeable reactive barriers can also be an interesting option for simultaneously removing anionic and cationic ions from aqueous media, each one using a material with a different pH_pzc_^16,17^.

#### Influence of the S:L ratio

**Figure 5:**
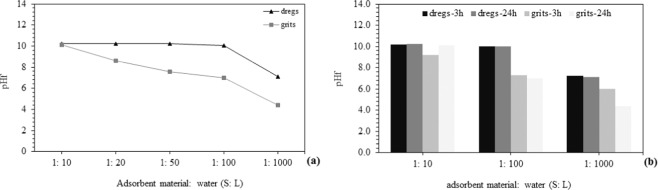
Buffer capacity of adsorption materials: (**a**) time: 3 h; (**b**) time: 3 h and 24 h.

The influence of the S:L ratio is depicted in Fig. 5a,b. Both materials exhibit alkaline properties, even when high S:L ratios are tested. Namely, for dregs, the pH was approximately 10 until the S:L is equal to 1:100. For an S:L ratio of 1:1000, the pH was always above 7 (Fig. 5a). In this pH condition, some metals (e.g., Cu) may precipitate and thus be removed from the liquid^52^. A significant decrease of the pH for the dregs batch adsorption test was observed for S:L higher than 1:100, but the values were similar for assays after 3 h and 24 h. The behavior of grits is slightly different (Fig. 5b). Chemical precipitation is the mechanism traditionally explored for metal removal in AMD^60^, but this process has been questioned due to the high amount of alkali needed, such as limestone^61^. For this reason, the use of alternative materials, such as dregs and grits, may be advantageous, due to the large availability and low cost.

### Adsorption kinetics

Figure 4 shows the adsorption kinetics, starting with initial concentrations of Cu and SO_4_ of 600 mg/L and 4,300 mg/L, respectively. Figure 4a reveals a maximum removal of 63% (189 mg/g of dregs) and 80% (240 mg/g of grits) for Cu, and Fig. 4b shows a removal of 79% (1,699 mg/g of dregs) and 89% (1,914 mg/g of grits) for SO_4_. The maximum removal (minimum concentration) for Cu occurred at 5 min with dregs and 180 min with grits. The maximum removal for SO_4_ occurred at 60 min for dregs and at 90 min for grits.

Figure 4c reveals a reduction of 99.5% (299 mg/g) of Cu concentration at 5 min using dregs, without pH control (alkaline pH). A significant Cu removal with grits occurred after a longer time, and at 180 min achieved a reduction of 97%. The fast and high retention of Cu by dregs suggest its precipitation in Cu(OH)_2_, common in alkaline conditions^62^. A sulfate retention of 28% using both materials was achieved during the kinetic assay without pH control. Both precipitation and adsorption mechanisms seem to have an important role according to these results. Chemical precipitation was also found to be the main mechanism for Pb removal from underground water using wastewater treatment plant sludge as PRB^63^. Other studies from the literature reported fast kinetics. For example, the maximum adsorption of Ni and Cu was 5 min and 15 min, respectively, using MgO as adsorbent material^64^. Also, the retention of a synthetic solution of Cu with an initial concentration of 400 mg/L showed removal within 30 min, using calcium silicate powder as adsorbent^65^. For a synthetic solution of sulfate with an initial concentration of 1,800 mg/L and pH 4.5, removal higher than 85% occurred at 10 min, using AlCl_3_ as an adsorbent material, by a co-precipitation mechanism^9^. The selective precipitation of copper occurs with a pH in the range of 2.8–4.7 and increases over time^52^. A similar precipitation mechanism was observed using a molar rate of 1:1 of calcium carbonate and Cu, presenting more than 99% Cu removal^66^.

### Adsorption batch tests

**Figure 6:**
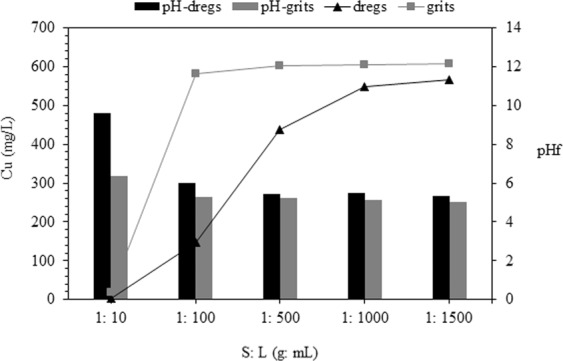
Copper adsorption for dregs and grits for different S:L ratios.

Removal of Cu from liquid was 99% and 97% for dregs and grits, respectively, using an S:L ratio of up to 1:10 (Fig. 6). The Cu removal values decreased from 75% to 27% as the S:L ratio was raised from 1:100 to 1:500 in dregs treatment. Regarding grits, only 3% efficiency was observed for an S:L ratio equal to or greater than 1:100.

**Figure 7:**
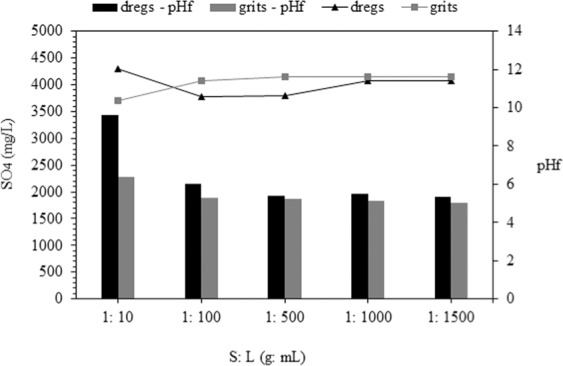
Sulfate concentration for different S:L ratios.

The initial sulfate concentration was 4,300 mg/L and the removal efficiency for dregs and grits ranged from 5–12% and 14–13.5%, respectively (Fig. 7). The removal efficiency was reduced as the S:L ratio decreased. However, this variation was not significant. Sulfate is influenced indirectly by pH variation, since in acidic media (pH < 2) there is an increase in the metal concentrations in solution. This may cause a decrease in the sulfate concentration by its precipitation, such as in the case of FeSO_4_^67^.

### Adsorption in column tests

**Figure 8:**
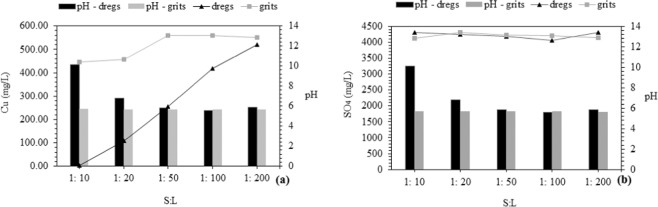
Copper (**a**) and sulfate (**b**) removed in columns filled with dregs and grits.

The continuous up-flow column test in the laboratory was used to simulate the reactive effect of the adsorbent materials intended to be used as a permeable reactive barrier (PRB)^3,17,21,68,69^. Figure 8 shows the results obtained using a column setup, until 200 mL per each gram of dregs and grits in the column. Cu retention was 99%, 82% and 56% using dregs with an S:L ratio of 1:10, 1:20 and 1:50, respectively. For grits, these values were only 26%, 24% and 7% for the same ratios, respectively (Fig. 8a). The sulfate removal was below 6% for all adsorption materials and S:L ratios (Fig. 8b). The high Cu removal was verified for alkaline pH, suggesting the precipitation mechanism. In this condition, at a 1:20 (solid:liquid) ratio, there will be a consumption of 0.05 t of material to treat a cubic meter of acid mine drainage (AMD). The quantity of material and lifespan of the PRB can be estimated through modeling techniques^14^. For instance, in the case of an abandoned gold and silver mine in Japan with an AMD flow of 18,000 m³/year^70^, it would take approximately 900 t/year of dregs or grits to treat the drainage. The mining activity in Minas Gerais state in Brazil has a potential for generating 104 billion cubic meters of AMD annually, with a cost of US$ 26 million for using commercial limestone in AMD remediation^71^.

The recovery of rare earth elements from coal, through the use of permeable reactive barrier material as evaluated in the present study, can be obtained through solvent extraction, electrowinning, magnetic and electrostatic separation, flotation or precipitation^72^. This aspect can be an opportunity that adds value to the PRB, which should be studied in the future.

## Conclusions

Dregs and grits showed a potential for being used as permeable reactive barriers for Cu removal due to their alkaline characteristics, based on their high calcium oxide content. The main Cu removal mechanism was chemical precipitation, notably for the S:L ratio of 1:10, when a removal above 99% was obtained.

The sulfate removal efficiency occurred at pH below 5, demanding about 18 mmol H^+^ (HCl) per gram of dregs and grits.

The high values of the point of zero charge (pH_pzc_) for dregs e grits, due to the presence of carbonates and hydroxides, suggest a potential for adsorption of anionic ions such as sulfates. Moreover this characteristic benefits the chemical precipitation of metals such as copper.

The high porosity of these materials, specially dregs, helps the contaminant removal from water by means of inter-particles diffusion.

Therefore, three potential mechanisms were observed in the removal of sulfate and copper: adsorption, chemical precipitation and inter-particle diffusion.

Future studies should use of dregs and grits treating real mine drainage solution.
